# Epidemiology survey and risk factor analysis of overactive bladder in children in Qingdao, China

**DOI:** 10.1016/j.jped.2026.101558

**Published:** 2026-05-23

**Authors:** Dong Yan, Xiang Li, Lei Zhang, Xian Yang, Qi Zhou, Chao Sun, Jing Li, Chao Liu

**Affiliations:** a Qilu Hospital of Shandong University (Qingdao), Department of Anesthesia, Qingdao, China; b Qilu Hospital of Shandong University (Qingdao), Department of Pediatric Surgery, Qingdao, China; c Qilu Hospital of Shandong University, Department of Pediatric Surgery, Jinan, China; d Qilu Hospital of Shandong University (Qingdao), Department of Pediatrics, Qingdao, China

**Keywords:** Overactive bladder, Children, Epidemiology survey, Risk factors, Qingdao

## Abstract

**Objective:**

Explore the epidemiological characteristics and risk factors of overactive bladder (OAB) in children in Qingdao, China.

**Method:**

A stratified random cluster sampling method was adopted from 20 primary schools of Qingdao between September 2024 and June 2025. Data were collected through anonymous questionnaires, including basic information, lower urinary tract symptoms, Overactive Bladder Symptom Score, Pediatric Sleep Questionnaire (PSQ) and Strengths and Difficulties Questionnaire. Meanwhile, body mass index (BMI), history of nocturnal enuresis (NE), diaper use, urinary tract infection, constipation, and external genitalia development were collected.

**Results:**

3200 questionnaires were distributed, with 2986 valid ones recovered, including 1492 boys and 1494 girls. The morbidity of OAB was 5.86% (175/2986), with 7.24% (108/1492) in boys and 4.46% (67/1494) in girls, and the difference was statistically significant (P < 0.001). The morbidity of children over 9-year-old (4.45%, 77/1683) and those under 9-year-old (7.52%, 98/1303) was statistically significant (P < 0.001). NE history, urinary tract infection history, constipation, excessive use of diapers, boy with phimosis or redundant prepuce were independent risk factors. The scores of emotional symptoms, conduct problems, hyperactivity symptoms, peer relationship problems, total difficulty score, and total PSQ score in the OAB group were significantly higher than those of the non-OAB group, with statistically significant differences (all P < 0.05).

**Conclusions:**

Boys and children under 9-year-old are at higher risk of OAB in Qingdao. History of NE, urinary tract infection, constipation, excessive use of diapers, and a boy with phimosis or redundant prepuce are the main independent risk factors. Meanwhile, OAB significantly affects children's mental health and sleep quality.

## Introduction

Overactive Bladder (OAB) is defined by the International Continence Society (ICS) as a bladder dysfunction characterized by urgency as the core symptom, with or without urge urinary incontinence, often accompanied by frequent micturition and nocturia, and without urinary tract infection or other clear organic lesions [1]. Long-term symptoms such as urgency and frequent micturition are prone to cause children to experience urinary leakage and wet clothes, leading to teasing by peers and reduced self-identity, thereby affecting their social activities and mental health. Frequent urination can also interfere with classroom learning rhythm, reduce learning efficiency, and cause dual negative impacts on children's physical and mental development [2]. Previous literature reports that the prevalence of OAB in non-Chinese children is 5%−16.59% [3,4]. Domestic related studies in China are mostly concentrated in inland areas. The prevalence of OAB in boys in Henan Province is 6.0% [5], while epidemiological data on OAB in children in developed coastal cities of China are still lacking, and the region-specific risk factors have not been clarified. Qingdao has differences in children's living environment, dietary structure, and parenting methods compared with inland areas. Conducting an epidemiological survey of childhood OAB in Qingdao has important practical significance.

This study systematically analyzes the prevalence rate, age and gender distribution characteristics of OAB in children in Qingdao, and deeply explores the risk factors affecting its occurrence and the impact on children's psychological behavior and sleep quality, in order to provide data support for formulating child OAB prevention strategies in line with regional characteristics and improving the level of children's urinary system health.

## Methods

### Study population

A multi-stage stratified random cluster sampling method was used to select study population from the 7 administrative districts of Qingdao between September 2024 and June 2025. 20 schools were selected from 7 administrative districts. Stratification was carried out by grade, and one class was randomly selected from each grade.

This study was approved by the Medical Ethics Committee of Qilu Hospital of Shandong University (Qingdao) (approval number: KYLL-KS-2025238).

Inclusion criteria: (1) Children living in Qingdao continuously for >1 year. (2) The parents are informed of the content to be surveyed in this study and voluntarily participate in this study. (3) No history of neurological diseases and congenital urinary system malformations. (4) No severe organic diseases such as heart, liver, and kidney, no immune system diseases, and no metabolic diseases.

Exclusion criteria: (1) Secondary urgency and frequent micturition symptoms caused by organic diseases. (2) Incomplete questionnaire filling (missing key information exceeding 10%) or logical contradictions. (3) Acute urinary tract infection during the survey.

## Research methods

### Survey tools

Referring to the “Chinese Expert Consensus on the Diagnosis and Treatment of Pediatric Overactive Bladder” [6], the research team designed the “Questionnaire on Bladder Function and Related Factors of Children” independently. A pre-survey (with a sample size of 50 cases from different ages in a school) showed that the Cronbach's α coefficient of the questionnaire was 0.86, and the content validity index was 0.88, indicating good reliability and validity of the questionnaire. The questionnaire includes four aspects.

#### Basic information

Gender, age, administrative district, parents' educational level, and family monthly income.

#### OAB-related symptoms and scores

The Overactive Bladder Symptom Score (OABSS) was used to evaluate the severity of symptoms. The score includes 4 dimensions (daytime micturition frequency, nocturnal micturition frequency, urgency frequency, and urge urinary incontinence frequency), with a total score of 0–15 points. The diagnostic criteria for OAB are urgency score ≥ 2 points and total score ≥ 3 points. A total score of ≤ 5 points is mild, 6–11 is moderate, and ≥ 12 is severe [7].

#### Potential risk factors

(1) Diaper use. Whether to continue using diapers after 3 years old. (2) Past medical history. Whether there is a history of nocturnal enuresis (NE, involuntary nocturnal urination ≥ 2 times a week for >3 months) and urinary tract infection diagnosed by a hospital in the past. (3) Physical condition. constipation (defecation ≤ 2 times a week or difficulty in defecation, dry stool for >1 month), body mass index (BMI). Determine the BMI range in accordance with the Screening for Overweight and Obesity in School-Age Children and Adolescents (Health Industry Standard of the People's Republic of China, WS/T 586—2018) [8]. (4) External genitalia development of boys. Whether combined with phimosis or redundant prepuce.

#### Psychological behavior and sleep assessment

The Strengths and Difficulties Questionnaire (SDQ) was used to evaluate psychological and behavioral problems. The questionnaire includes 5 dimensions, including emotional symptoms, conduct problems, hyperactivity symptoms, peer relationship problems, and prosocial behavior. The total difficulty score is the sum of scores of the first 4 dimensions. 0–15 points is normal, 16–19 is suspicious, and >20 is abnormal [9]. The Pediatric Sleep Questionnaire (PSQ) was used to evaluate sleep quality [10]. The total score is obtained by calculating the average of all item scores. A higher score indicates worse sleep quality [11].

### Survey implementation

Parents were informed about the purpose of the survey and the method of filling out the questionnaire. All anonymous online questionnaires must undergo strict inspection and verification. For children whose OABSS score meets the diagnostic criteria for OAB, doctors conducted outpatient urine examination and ultrasound examination to exclude urinary tract infection or bladder structural abnormalities, and finally confirmed OAB cases.

### Statistical methods

Epi Data 3.1 software was used for double-entry and data verification by two persons, and SPSS 28.0 software was used for statistical analysis. Count data were expressed as frequency (n) and percentage (%), and inter-group comparison was performed using the chi-square test. Measurement data were expressed as (M ± SD), and inter-group comparison was performed using an independent sample *t*-test. Factors with P < 0.1 in univariate analysis were included in multivariate logistic regression analysis (with whether to have OAB as the dependent variable, assignment: yes = 1, no = 0; each risk factor as the independent variable, assignment according to variable type) to screen independent risk factors for OAB. P < 0.05 was considered statistically significant.

## Results

### Prevalence rate and distribution characteristics

A total of 3200 questionnaires were distributed, and 2986 valid questionnaires were recovered, with an effective recovery rate of 93.31%. There were 1492 boys (49.97%) and 1494 girls (50.03%). 1303 children aged under 9-year-old (43.63%) and 1683 children aged over 9-year-old (56.37%).

175 children were diagnosed with OAB, with an overall prevalence rate of 5.86% (175/2986). According to the OABSS score, there were 62 cases of mild OAB (35.43%, 62/175), 112 cases of moderate (63.99%, 112/175), and 1 case of severe (0.57%, 1/175), with no extremely severe cases. There were 108 boys with a prevalence rate of 7.24% (108/1492) and 67 girls with a prevalence rate of 4.46% (67/1494) among the children with OAB. The incidence rate of boys is significantly higher than that of girls (χ² = 10.236, P < 0.001).

Among OAB children, 98 were under 9-year-old, with a prevalence rate of 7.52% (98/1303), while 77 were over 9-year-old, with a prevalence rate of 4.45% (77/1683).

The prevalence rate in the under 9-year-old group was significantly higher (χ² = 14.872, P < 0.001). There was no statistically significant difference in the prevalence rate of OAB children in 7 administrative districts (χ² = 3.215, P = 0.781), as shown in Table 1.

**Table 1:** Distribution characteristics of OAB prevalence rate in children in Qingdao (n,%).

Characteristics	Total Cases	OAB Cases	Prevalence Rate (%)	χ² Value	P Value
Gender				10.236	<0.001
Boy	1492	108	7.24		
Girl	1494	67	4.46		
Age (years)				14.872	<0.001
<9	1303	98	7.52		
≥9	1683	77	4.45		
Administrative Region				3.215	0.781
Shinan	230	12	5.21		
Shibei	457	28	6.13		
Laoshan	220	11	5.00		
Licang	311	18	5.78		
Chengyang	759	48	6.32		
Huangdao	786	47	5.98		
Jimo	194	11	5.67		

### Univariate analysis of OAB occurrence

A univariate chi-square test was performed on 10 factors. The results showed that gender (boy), age (under 9-year-old), NE history, urinary tract infection history, constipation, excessive use of diapers, phimosis (boy), and redundant prepuce (boy) were associated with the occurrence of OAB (all P < 0.05), while parents' educational level, family monthly income, and BMI were not significantly associated with OAB in children (all P > 0.05), as shown in Table 2.

**Table 2:** Univariate analysis of OAB occurrence in children in Qingdao (n, %).

Factors	Categories	Total Cases	OAB Cases	Prevalence Rate (%)	χ² Value	P Value
Gender					10.236	<0.001
	Boy	1492	108	7.24		
	Girl	1494	67	4.46		
Age (years)					14.872	<0.001
	<9	1303	98	7.52		
	≥9	1683	77	4.45		
Parents' Educational Level					0.321	0.852
	High school or below	892	52	5.83		
	College	1126	66	5.86		
	Postgraduate or above	968	57	5.89		
Family Monthly Income (¥, yuan)					0.287	0.866
	<10,000	956	56	5.86		
	10,000∼25,000	1235	72	5.83		
	>25,000	795	47	5.91		
NE History					78.325	<0.001
	Yes	186	42	22.58		
	No	2800	133	4.75		
Urinary Tract Infection History					95.672	<0.001
	Yes	72	24	33.33		
	No	2914	151	5.18		
Constipation					102.458	<0.001
	Yes	215	52	24.19		
	No	2771	123	4.44		
Excessive Use of Diapers					9.763	0.002
	Yes	1286	98	7.62		
	No	1700	77	4.53		
BMI					0.892	0.640
	Normal	2015	118	5.86		
	Overweight	658	38	5.78		
	Obese	313	19	6.07		
Phimosis (Boy)					45.892	<0.001
	Yes	215	38	17.67		
	No	1277	70	5.48		
Redundant Prepuce (Boy)					68.325	<0.001
	Yes	186	45	24.19		
	No	1306	63	4.82		

### Multivariate logistic regression analysis of OAB occurrence

Eight factors with P < 0.1 in univariate analysis were included in the multivariate logistic regression model. The results showed that NE history, urinary tract infection history, constipation, excessive use of diapers, phimosis, and redundant prepuce were independent risk factors for OAB (all P < 0.05), while gender and age were not independent risk factors (all P > 0.05), as shown in Table 3.

**Table 3:** Multivariate logistic regression analysis of OAB occurrence in children in Qingdao.

Factors	Assignment	Regression Coefficient	Standard Error	P Value	OR Value	95%CI
Gender	Boy=1, Girl=0	0.286	0.175	0.105	1.331	0.945∼1.878
Age (years)	<9 = 1, ≥9 = 0	0.215	0.168	0.203	1.240	0.887∼1.733
NE History	Yes=1, No=0	1.728	0.296	<0.001	5.623	3.215∼9.837
Urinary Tract Infection History	Yes=1, No=0	2.189	0.291	<0.001	8.912	5.126∼15.478
Constipation	Yes=1, No=0	1.918	0.205	<0.001	6.815	4.628∼9.983
Excessive Use of Diapers	Yes=1, No=0	0.545	0.178	0.001	1.725	1.236∼2.411
Phimosis (Boy)	Yes=1, No=0	0.973	0.185	<0.001	2.638	1.845∼3.769
Redundant Prepuce (Boy)	Yes=1, No=0	1.418	0.183	<0.001	4.126	2.903∼5.867

### Comparison of psychological behavior and sleep quality between OAB group and non-OAB group

The scores of emotional symptoms, conduct problems, hyperactivity symptoms, peer relationship problems, total difficulty score, and total PSQ score in the OAB group were significantly higher than the non-OAB group, with statistically significant differences (all P < 0.05), and there was no statistically significant difference in prosocial behavior score between the two groups (P > 0.05), as shown in Table 4.

**Table 4:** Comparison of psychological behavior and sleep quality between OAB group and non-OAB group in children in Qingdao (M ± SD).

Indicators	OAB Group (n = 175)	Non-OAB Group (n = 2811)	t Value	P Value
Emotional Symptom Score	7.58±2.11	6.48±2.55	−4.987	<0.001
Conduct Problem Score	8.09±1.65	7.29±1.89	−4.892	<0.001
Hyperactivity Symptom Score	5.59±2.31	4.96±2.05	−3.215	0.001
Peer Relationship Problem Score	7.12±1.68	6.57±1.62	−3.986	<0.001
Prosocial Behavior Score	3.12±1.92	3.23±2.01	−0.678	0.498
Total Difficulty Score	30.18±6.52	27.65±6.46	−4.478	<0.001
Total PSQ Score	5.32±3.05	3.92±2.55	−4.913	<0.001

## Discussion

### Epidemiological characteristics of OAB in children in Qingdao

The results showed that the overall prevalence rate of OAB in children in Qingdao was 5.86%, which was at the low end of the range of 5%−16.59% reported abroad [3], close to the prevalence rate of OAB in boys in a certain domestic area of China (6.0%) [5], but lower than the total prevalence rate of OAB in five provinces in China (9.01%) [12]. This difference may be related to the age range of the research subjects, the level of regional economic development, and the strictness of the implementation of diagnostic criteria.

In terms of gender, the prevalence rate of OAB in boys (7.24%) was significantly higher than girls (4.46%), which was consistent with the conclusions of partial studies [5,13,14]. It is speculated that the possible reasons including the urethra of boy is longer, the resistance at the bladder outlet is relatively higher, and boys have more outdoor activities, which are prone to delayed urination and holding urine, increasing the risk of unstable detrusor contraction [15]. The unique abnormal external genitalia of boys may also aggravate OAB symptoms by stimulating the urethra, which has been verified in the multivariate analysis of this study.

In terms of age, the prevalence rate of OAB in the under-9-year-old group (7.52%) was significantly higher than the over-9-year-old group (4.45%), which is closely related to the developmental law of children's bladder. The bladder capacity of under 9-year-old children is not yet mature (average capacity about 250–300 ml), and their perception of urination signals and voluntary control ability are weak, making it difficult to form regular urination habits, so they are more prone to symptoms such as urgency and frequent micturition [16], while with the improvement of nervous system development and the increase of bladder capacity (average capacity about 350–400 ml) in over 9-year-old children, their ability to regulate bladder function is enhanced, and the risk of OAB is reduced accordingly.

In terms of distribution in various administrative districts, there was no significant difference in the prevalence rate of OAB between children in the downtown of Qingdao and the suburbs (P = 0.781). This may be due to the rapid economic development of the suburbs of Qingdao in recent years, and children's lifestyles (such as dietary structure, academic pressure) have gradually converged with those in the downtown, resulting in no significant difference in the risk of OAB.

### Analysis of risk factors for OAB

#### NE history and urinary tract infection history

Multivariate analysis of this study showed that NE history was an independent risk factor for OAB (OR=5.623, P < 0.001). Children with NE are often accompanied by involuntary contraction of the bladder detrusor. This abnormal bladder function may persist, leading to OAB symptoms such as urgency and frequent micturition in children during the day [17]. Children with NE have poor nighttime urination control ability, which may further aggravate bladder dysfunction and increase the risk of OAB. A history of urinary tract infection is also an important independent risk factor (OR=8.912, P < 0.001). During a urinary tract infection, bacteria and their metabolites can stimulate the bladder mucosa, leading to increased sensitivity of the bladder detrusor and involuntary contraction. Even after the infection is cured, the inflammatory damage to the bladder mucosa may persist, leading to abnormal detrusor function and further development of OAB [18]. Clinical data show that the prevalence rate of OAB in children with a history of urinary tract infection (33.33%) is 6.4 times that of children without a history of infection (5.18%), suggesting that clinical attention should be paid to the standardized treatment of urinary tract infections in children to reduce the risk of OAB.

#### Constipation

The results of this study showed that constipation was an independent risk factor for OAB (OR = 6.815, P < 0.001). When children have constipation, dry stool will compress the bladder, leading to reduced bladder capacity and increased pressure, thereby stimulating the contraction of the bladder detrusor and causing symptoms such as urgency and frequent micturition. Meanwhile, an abnormal intestinal-bladder reflex may lead to insufficient inhibition of bladder activity by the cortical center, further aggravating detrusor overactivity [19]. In this study, the prevalence rate of OAB in children with constipation (24.19%) was 5.4 times that of normal children (4.44%), suggesting that clinically, when treating childhood OAB, it is necessary to simultaneously evaluate and improve constipation, such as relieving constipation through dietary adjustments (increasing dietary fiber intake) and regular defecation training, to improve the therapeutic effect of OAB.

#### Excessive use of diapers

Excessive use of diapers was an independent risk factor for OAB (OR = 1.725, P = 0.001). The bladder function of children over 2 years old gradually matures. Excessive use of diapers will make children lack perception of urination signals and voluntary control training, leading to decreased ability to regulate bladder urine storage function. The continuous wrapping of diapers may increase the temperature and humidity of the perineum, stimulate the urethra and bladder mucosa, and increase the risk of OAB [20]. In this study, the prevalence rate of OAB in children with excessive use of diapers (7.62%) was higher than that in children with normal use (4.53%), suggesting that parents should timely conduct toilet training for children around 3 years old, reduce the use of diapers, and promote the normal development of bladder function.

#### Phimosis and redundant prepuce

Analysis of boys in this study showed that phimosis (OR = 2.638, P < 0.001) and redundant prepuce (OR = 4.126, P < 0.001) were independent risk factors for OAB. Phimosis and redundant prepuce can lead to narrowing of the prepuce orifice or accumulation of prepuce smegma, stimulating the external urethral orifice and glans penis, causing congestion and edema of the urethral mucosa, which is then transmitted to the bladder, leading to increased sensitivity of the bladder detrusor and causing OAB symptoms such as urgency and frequent micturition [21]. Redundant prepuce may also increase the risk of urinary tract infection, indirectly aggravating OAB symptoms. In this study, the prevalence rate of OAB in boys with phimosis (17.67%) was 3.2 times that of normal (5.48%), and boys with redundant prepuce (24.19%) was 5.0 times that of normal (4.82%), suggesting that clinical attention should be paid to the evaluation of external genitalia development in boys, and timely intervention should be performed for children with phimosis and redundant prepuce to reduce the risk of OAB.

#### BMI was not an independent risk factor

Univariate analysis of this study showed that BMI was not significantly associated with the occurrence of OAB (P = 0.640). Previous studies have suggested that obesity may affect bladder function by compressing pelvic organs [22], but this study did not find an association between BMI and OAB. This may be due to the mild obesity of children (the proportion of obese children in this study was 10.48%), and the compression effect on the bladder is not obvious. Children are in the stage of growth and development, and their body structure is different from that of adults, so the impact of obesity on bladder function may be different from that of adults [23]. Literature has shown that in recent years, the obesity rate among urban children in this region has been higher than that in rural areas, and the childhood obesity rate has shown an upward trend. With the rise in the obesity rate, the incidence of overactive bladder (OAB) may increase [24].

### Impact of OAB on children's psychological behavior and sleep quality

The results of this study showed that the scores of emotional symptoms, conduct problems, hyperactivity symptoms, peer relationship problems, total difficulty score, and total PSQ score in the OAB group were significantly higher than those in the non-OAB group (all P < 0.05). Children with OAB are prone to urinary leakage and wet clothes due to urgency and frequent micturition, which may be teased by peers, leading to reduced self-identity and emotional problems such as anxiety and depression. Frequent toilet visits can interfere with classroom learning and outdoor activities, leading to behavioral problems such as inattention and hyperactivity in children, further affecting peer relationships [25]. In terms of sleep quality, children with OAB have an increased number of nocturnal urinations, which may wake up frequently, leading to sleep interruption and reduced sleep quality. Insufficient sleep may further aggravate emotional and behavioral problems, forming a vicious circle [26]. Clinical data show that the total PSQ score of children in the OAB group (5.32 ± 3.05) is 1.36 times that of the non-OAB group (3.92 ± 2.55), suggesting that, clinically, while treating OAB symptoms, attention should be paid to children's mental health and sleep quality, and psychological intervention and sleep guidance should be performed when necessary.

At present, there are no fewer than 700,000 primary school students in Qingdao, and the number of students in this sampling survey only accounts for about 0.4%. The incidence rate and influencing factors in the research results may not represent the whole. In addition, this study did not analyze the treatment methods and effects of OAB, which has certain limitations.

In conclusion, boys and children under 9-year-old in Qingdao have a higher risk of OAB. NE history, urinary tract infection history, constipation, excessive use of diapers, and combined phimosis or redundant prepuce in boys are the main independent risk factors, and OAB can significantly affect children's mental health and sleep quality. Clinically, targeted comprehensive prevention and treatment measures should be carried out, such as strengthening the standardized treatment of urinary tract infections in children, improving constipation, guiding parents to use diapers scientifically, and timely intervening in abnormal external genitalia of boys. Meanwhile, attention should be paid to the psychological and sleep problems of children with OAB to reduce the risk of OAB in children and improve their health level.

## Authors’ contributions

XL and DY have contributed equally to this work and share first authorship. XL, DY and CL: the conception and design of the study. XL, LZ, XY, QZ, CS, JL and CL: acquisition of data. DY, LZ and CL: analysis and interpretation of data. XL, DY and CL: drafting the article. CL: revising it critically for important intellectual content. All authors approved the final version of the manuscript.

## Funding

None.

## Data availability

The datasets generated and analyzed during the current study are not publicly available due to restrictions on the disclosure of research data by the research office of the studied hospital, but are available from the corresponding author on reasonable request.

## Conflicts of interest

The authors declare no conflicts of interest.
